# Hybrid SPECT/CT for the assessment of a painful hip after uncemented total hip arthroplasty

**DOI:** 10.1186/s12880-015-0056-1

**Published:** 2015-06-02

**Authors:** Oliver Dobrindt, Holger Amthauer, Alexander Krueger, Juri Ruf, Heiko Wissel, Oliver S Grosser, Max Seidensticker, Christoph H Lohmann

**Affiliations:** Klinik für Radiologie und Nuklearmedizin, Universitätsklinikum Magdeburg A.ö.R., Otto-von-Guericke Universität, Leipziger Straße 44, 39120 Magdeburg, Germany; Charité - Universitätsmedizin, Klinik für Orthopädie, Centrum für Muskuloskeletale Chirurgie, Charitéplatz 1, 10117 Berlin, Germany; Orthopädische Universitätsklinik, Universitätsklinikum Magdeburg A.ö.R., Otto-von-Guericke Universität, Leipziger Straße 44, 39120 Magdeburg, Germany; Universitätsklinikum Freiburg, Klinik für Nuklearmedizin, Hugstetter Straße 55, 79106 Freiburg, Germany

**Keywords:** SPECT/CT, Total hip arthroplasty, THR, Hip pain, Loosening

## Abstract

**Background:**

The diagnosis of hip pain after total hip replacement (THR) represents a highly challenging question that is of increasing concern to orthopedic surgeons. This retrospective study assesses bone scintigraphy with Hybrid SPECT/CT for the diagnosis of painful THR in a selected cohort of patients.

**Methods:**

Bone SPECT/CT datasets of 23 patients (mean age 68.9 years) with a painful hip after THR were evaluated. Selection of the patients required an inconclusive radiograph, normal serum levels of inflammatory parameters (CRP and ESR) or a negative aspiration of the hip joint prior to the examination. The standard of reference was established by an interdisciplinary adjudication-panel using all imaging data and clinical follow-up data (>12 month). Pathological and physiological uptake patterns were defined and applied.

**Results:**

The cause of pain in this study group could be determined in 18 out of 23 cases. Reasons were aseptic loosening (n = 5), spine-related (n = 5), heterotopic ossification (n = 5), neuronal (n = 1), septic loosening (n = 1) and periprosthetic stress fracture (n = 1). In (n = 5) cases the cause of hip pain could not be identified. SPECT/CT imaging correctly identified the cause of pain in (n = 13) cases, in which the integrated CT-information led to the correct diagnosis in (n = 4) cases, mainly through superior anatomic correlation. Loosening was correctly assessed in all cases with a definite diagnosis.

**Conclusions:**

SPECT/CT of THA reliably detects or rules out loosening and provides valuable information about heterotopic ossifications. Furthermore differential diagnoses may be detected with a whole-body scan and mechanical or osseous failure is covered by CT-imaging. SPECT/CT holds great potential for imaging-based assessment of painful prostheses.

## Background

Total hip arthroplasty (THA) performed as primary intervention or revision is in both cases a procedure with increasing application and prevalence [1]. Problems associated will therefore increasingly engage surgeons and diagnosticians. The most common reasons for revision of THA are aseptic loosening (71.8%), dislocation (8.8%), periprosthetic infection (8.1%), fracture (6.9%), technical error (2.2%), implant fracture (1.5%), pain only (0.4%) and miscellaneous (0.3%) [2]. Causes of hip pain that are not directly related to the hip joint may be due to a local neurological or vascular pathology, inguinal hernia, metastatic cancer or spinal pathology with radiculopathy and radiating pain [3]. Determination of the pain etiology is of paramount importance for subsequent treatment [4].

With the technical advances and increasing availability of SPECT/CT over the last decade, new opportunities have opened up to further improve the quality of diagnosis in the field of orthopedic surgery [5-7]. White blood cell and anti-granulocyte scintigraphy with SPECT and integrated CT has already been evaluated for periprosthetic infections and delivered promising results showing improved anatomic mapping and improved specificity [8,9]. The aim of this clinical study was the assessment of bone scintigraphy with SPECT/CT for the determination of pain etiology after THA in a well-selected cohort of patients.

### Material and methods

This retrospective analysis has been reviewed and approved by the local ethics committee (University Magdeburg, Germany; assigned vote number 92/12) and was performed in accordance to the declaration of Helsinki.

### Patients and treatment

This study included 23 consecutive patients after total hip arthroplasty (male, n = 16; female, n = 7; mean age, 68.9 years (y)) who had bone scintigraphy with SPECT/CT for the assessment of hip pain within the clinical routine and according to clinical suspicion.

Inclusion criteria of the patients required an inconclusive conventional radiograph and non-elevated C-reactive protein (CRP) and erythrocyte sedimentation rate (ESR) values. In cases of elevated CRP (>5 mg/L) or ESR (>30 mm/hr) a negative aspiration of the symptomatic hip joint was required. The following information was obtained for each patient: age, sex, type of pain, localization, beginning of symptoms, type of implants, laboratory tests, microbiological results, radiographs, bone scintigraphy (BS) including SPECT/CT and magnet resonance imaging (MRI) if available.

### Imaging protocol

BS including SPECT/CT was performed with a hybrid gamma camera/CT (Discovery™ NM/CT 670, GE Healthcare) equipped with low-energy, high-resolution collimators. A three-phase BS was performed after the intravenous bolus-injection of 396-600 MBq (10.7-16.2 mCi) Tc-99 m DPD (TECEOS^®^; CIS bio international, Gif-sur-Yvette, France). Dynamic scintigraphy of the pelvis/hip region was recorded in a 128×128 matrix at a 2 second frame rate for the first minute in anterior-posterior orientation. Static blood-pool images were obtained in the following 3 minutes. For the mineralization phase (3 h after injection), planar images were acquired over 5 minutes in the same view. These acquisitions were performed in a double-head mode. In addition anterior and posterior whole-body scans were performed at a table speed of 13 cm/min using a 256×1024 matrix.

SPECT imaging with CT for attenuation correction of the hip/pelvis region and secondary reconstruction for SPECT/CT post processing in a single procedure was established. Projection data of SPECT were acquired at 140.5 keV +/- 10 % with an additional scatter window at 120.0 +/- 5 %, 60 views (20 s/view) over 360°, 128 × 128 matrix with a pixel size of 4.42 mm × 4.42 mm and with a body contoured scan orbit. Images were reconstructed by iterative reconstruction (2 iterations, 10 subsets) with a model-based resolution recovery algorithm (Evolution for Bone^®^, GE Healthcare). Reconstructed images were corrected for scatter and attenuation.

Low-dose CT was performed to provide an anatomical map for SPECT attenuation correction by a helical scan with an effective tube current of 40 mA, 120 kV, pitch 1.375, t_rot_ 0.8 s and 20.0 mm detector coverage (collimation: 1.25 mm × 16 slices). Secondary reconstruction of the CT raw data was performed to generate anatomical maps with higher resolution for registration with SPECT images. This advanced CT reconstruction with an isotropic voxel size of 1.25 mm was performed by the CT workstation (GE Healthcare, BrightSpeed™ Elite) using half-scan reconstruction (Pitch Booster™) and subsequently iterative CT reconstruction (Adaptive Statistical Iterative Reconstruction - ASiR™) for noise reduction. All imaging data were reviewed and processed on a Xeleris 3 workstation (GE Healthcare). An additional processing with multi-planar reconstruction of the registered SPECT/CT data was performed by OSIRIX MD 2.5.1 software (Pixmeo SARL, Bernex, Switzerland) to localize the bone/prosthesis interfaces on the anatomical map provided by the CT.

### Interpretation of imaging data

Imaging datasets consisted of a plain radiograph, a 3-phase BS including SPECT as well as integrated SPECT/CT of the hip and pelvis.

As the inclusion criteria required an inconclusive radiograph, it served as an anatomic map for the interpretation of bone scintigraphy. The perfusion images were visually analysed for the presence of increased tracer uptake in the sequential as well as the summation image. Blood pool images and images of the mineralization phase were also assessed for focal tracer uptake in the affected region.

For the detection or exclusion of loosening, a classification for normal, physiological and pathological uptake patterns for SPECT/CT was developed incorporating the crucial fixation areas of the acetabular and femoral components (Table 1).

**Table 1:** **Combined SPECT/CT criteria for the assessment of loosening of uncemented hip prostheses**

		**SPECT**	**CT**
**A - Acetabular component**	*1 - Normal*	No increased uptake	No radiolucency, no osteolysis
	*2 - Physiological remodeling*	Increased uptake of surrounding bone at the superior and/or inferior third of the cup	No radiolucency or osteolysis in areas corresponding to SPECT findings (differentiation of surrounding bone and prosthesis-bone interface)
	*3 - Pathological loosening*	1 - Increased uptake of the whole prosthesis-bone interface	Possibly radiolucency or osteolysis at the prosthesis-bone interface
		2 - Increased uptake of the intermediate third in combination with the inferior or superior third	Possibly radiolucency at the prosthesis-bone interface
		3 - Increased uptake in the superior or inferior third of the component	In combination with a radiolucent line or osteolysis in the area corresponding to SPECT findings
**F - Femoral component**	*1 - Normal*	No increased uptake	No radiolucency, no osteolysis
	*2 - Physiological remodeling*	1 - Increased uptake at the prosthesis-dependent zones not crucial for fixation	Possibly radiolucency at the prosthesis-bone interface, no radiolucency or osteolysis at crucial fixation zones
		2 - Increased uptake of surrounding bone at the crucial fixation zones	No radiolucency or osteolysis in corresponding areas (differentiation of surrounding bone and prosthesis-bone interface)
	*3 - Pathological loosening*	1 - Increased uptake of the whole prosthesis-bone interface	Possibly radiolucency or osteolysis at the prosthesis-bone interface
		2 - Multifocal uptake with at least one focus within the crucial fixation zone of the prosthesis	Possibly radiolucency or osteolysis at the prosthesis-bone interface

BS images were rated in a separate consensus reading by two specialists in nuclear medicine and orthopedics. The value of integrated SPECT/CT in comparison to BS including SPECT and plain radiographs was also determined. In addition, SPECT and planar whole-body scintigraphy were evaluated for uptake irregularities apart from the hip-joint to search for other potential causes for hip pain.

The analysis of the CT images included the positioning of the acetabular and femoral component, wear of the polyethylene liner, hardware failure, heterotopic ossifications and fractures.

### Standard of reference

A reference standard was created by presenting each case to an adjudication panel consisting of experts in the fields of orthopedic surgery, nuclear medicine, and radiology. All available data (clinical records and follow-up data of at least 12 months, radionuclide imaging, radiographs, MRI, microbiology, histology, and intraoperative findings) were taken into consideration for the standard of reference and a final diagnosis for the cause of hip pain was documented. First all cases were presented excluding SPECT/CT images or written reports. In a second session all Images were presented with the readers blinded to the history of the patient.

If a definite reason could not be determined and the cause of pain remained unknown, all further derivatives were marked as not applicable.

## Results

A total of 23 consecutive patients (16 male/ 7 female) with a mean age of 68.9 years (y) (46.8 y – 84.8 y) and hip pain after total hip arthroplasty (THA) were included in this retrospective study. The mean time after implantation was 4.3 y (median 3.5 y; IQR 1.6 y – 4.8 y). In 15 out of 23 patients pain commenced after a pain-free interval after surgery, 8 out of 23 patients reported persistent postoperative pain. 10 out of 23 patients also had THA on the contralateral side, which were all clinically inconspicuous and showed normal uptake patterns (A1,F1) according to our SPECT/CT grading scale (Table 1).

In 18 out of 23 patients the cause of hip pain after THA was determined by the end of the follow-up period, including aseptic aseptic loosening (n = 5), spine-related (n = 5), heterotopic ossification (n = 5), neuronal (n = 1), septic loosening (n = 1), and a periprosthetic stress fracture (n = 1).

In 13/23 patients diagnosis was confirmed at surgery. In one patient the acetabular and femoral component were replaced, in one patient only the acetabular component and in 3 patients only the femoral component of the prosthesis was exchanged. In 3 patients with spine-related pain radiating into the hip, lumbar decompression was performed. In 3 patients heterotopic ossifications were removed surgically. In the case of septic loosening, a 2-stage exchange was performed. The periprosthetic stress fracture of the femur advanced to a fracture and was surgically treated with osteosynthesis. The success of the surgical procedures was confirmed in all cases by clinical follow-up.

In 5/23 patients surgery was not indicated and the diagnosis was confirmed by clinical records and clinical follow-up. In 1 patient the discomfort was caused by a neurological disorder causing muscular dysbalance and gait abnormality. In 2 cases of heterotopic ossifications and in 2 cases of spine-related hip-pain, surgery was impossible due to contraindications or was declined by the patients.

In the remaining 5/23 patients a final diagnosis failed to determine the cause of pain. Of these cases the symptoms subsided under conservative treatment in 2 patients, whereas persisting pain with unclear aetiology was found in 3 patients. An overview on patient characteristics is given in Table 2.

**Table 2:** **Characteristics and clinical data of patients with a painful hip after THA**

**Pat. no.**	**Gender/age (y.)**	**Cause of pain**	**Cause of pain detected with planar scintigraphy + SPECT + radiograph**	**Cause of pain detected with SPECT-/CT**	**SPECT/CT classification**	**Add. Information through Hybrid SPECT/CT**	**Loosening**	**Standard of reference/verification**
1	M/54	Loosening of cup	Yes	Yes	A3,2/F2,1	no	True-pos.	Surgery
2	M/58	Unknown	n.a.	n.a	A2/F2,1	n.a	n.a.	Follow up/persisting pain
3	M/47	Spine	No	No	A1/F1	No	True-neg.	Follow up/MRI confirmation of herniated vertebral disc
4	M/73	HO	Yes	Yes	A1/F1	No	True-neg.	Follow up
5	M/76	Unknown	n.a.	n.a	A1/F2,1	n.a	n.a.	Follow up/pain free under conservative treatment
6	M/58	Loosening of stem	Yes	Yes	A1/F3,2	No	True-pos.	Surgery
7	M/64	Spine	No	No	A1/F1	No	True-neg.	Surgery/MRI spinal stenosis
8	F/84	Spine	No	Yes	A1/F1	Detection of facet joint arthrosis and spinal stenosis	True-neg.	Surgery
9	M/58	HO	No	Yes	A1/F1	Localisation and differentiation of suspected loosening	True-neg.	Follow up
10	M/77	Spine	No	No	A1/F2,1	No	True-neg.	Follow up/MRI degenerative lumbar scoliosis
11	F/73	HO	Yes	Yes	A1/F1	No	True-neg.	Surgery
12	M/80	Loosening of stem	Yes	Yes	A2/F3,1	No	True-pos.	Surgery
13	M/82	Neuronal	No	No	A1/F2,1	No	True-neg.	Follow up/neurological examinations
14	M/70	HO	No	Yes	A2/F1	Anatomic mapping and differentiation	True-neg.	Surgery
15	M/63	Unknown	n.a.	n.a.	A1/F1	n.a.	n.a.	Follow up/pain free under conservative treatment
16	F/74	HO	Yes	Yes	A1/F1	No	True-neg.	Surgery
17	M/76	Spine	No	No	A1/F2,1	No	True-neg.	Surgery, lumbar decompression and stabilization
18	F/48	Unknown	No	Yes	A1/F1	Antetorsion stem 45°; anteversion of cup 35° - malalignment of compnents and possible impingement	n.a.	Follow up/persisting pain, malalignment
19	F/80	Loosening of stem	Yes	Yes	A2/F3,1	Anatomic mapping with coexisting HO	True-pos.	Surgery
20	F/81	Loosening of stem and cerclage	No	Yes	A2/F3,2	Improved mapping	True-pos.	Surgery
21	M/68	Unknown	n.a.	n.a	A1/F1	n.a	n.a.	Follow up/persisting pain
22	M/59	Septic loosening	Yes	Yes	A1/F3,2	No	True-pos.	Surgery/loosening was diagnosed but infection later clinically
23	F/82	Periprosthetic stress fracture	Yes	Yes	A1/F1	No	True-neg.	Follow up, surgery

Abbreviations: *M*, male; *F*, female; *HO*, heterotopic ossifications; n.a., not applicable; True-pos., true positive; True-neg., true negative.

### Conventional X-ray, 3-phase bone scintigraphy and SPECT

The cause of pain in 9 out of 18 patients with a definite diagnosis could be determined using the imaging algorithms consisting of radiographs, planar 3-phase BS and SPECT. Prosthetic loosening (n = 5), heterotopic ossification (n = 3) and a stress fracture (n = 1) were identified. Prosthetic loosening as a separate objective was correctly excluded in 10 out of 12 patients and diagnosed in 5 out of 6 cases (false positive, n = 2; false negative, n = 1).

### Hybrid SPECT/CT

13 of the ultimately identified 18 reasons for hip pain were diagnosed by Hybrid SPECT/CT imaging: prosthetic loosening (n = 6), heterotopic ossification (n = 5), lumbar spinal stenosis with facet joint arthrosis (n = 1) and a periprosthetic stress fracture (n = 1). One case of loosening was caused by a periprosthetic infection. This could not be differentiated from aseptic loosening and was not indicated by a significant increase of tracer uptake in the perfusion and blood pool phase. Arthritis of facet joints was initially seen by an increased uptake on the planar scintigraphy of the whole-body, and the field of view of the lumbo-pelvic SPECT/CT was adapted accordingly. Osseous hypertrophy of the facet joint was seen on the CT-scan and a consecutive radicular compression was verified via MRI before surgery was performed.

In the remaining 5 of the 18 patients SPECT/CT correctly ruled out a prosthesis associated pathology, which was confirmed by clinical and imaging follow-up (neurologic disorder n = 1; spine related n = 4).

**Figure 1:**
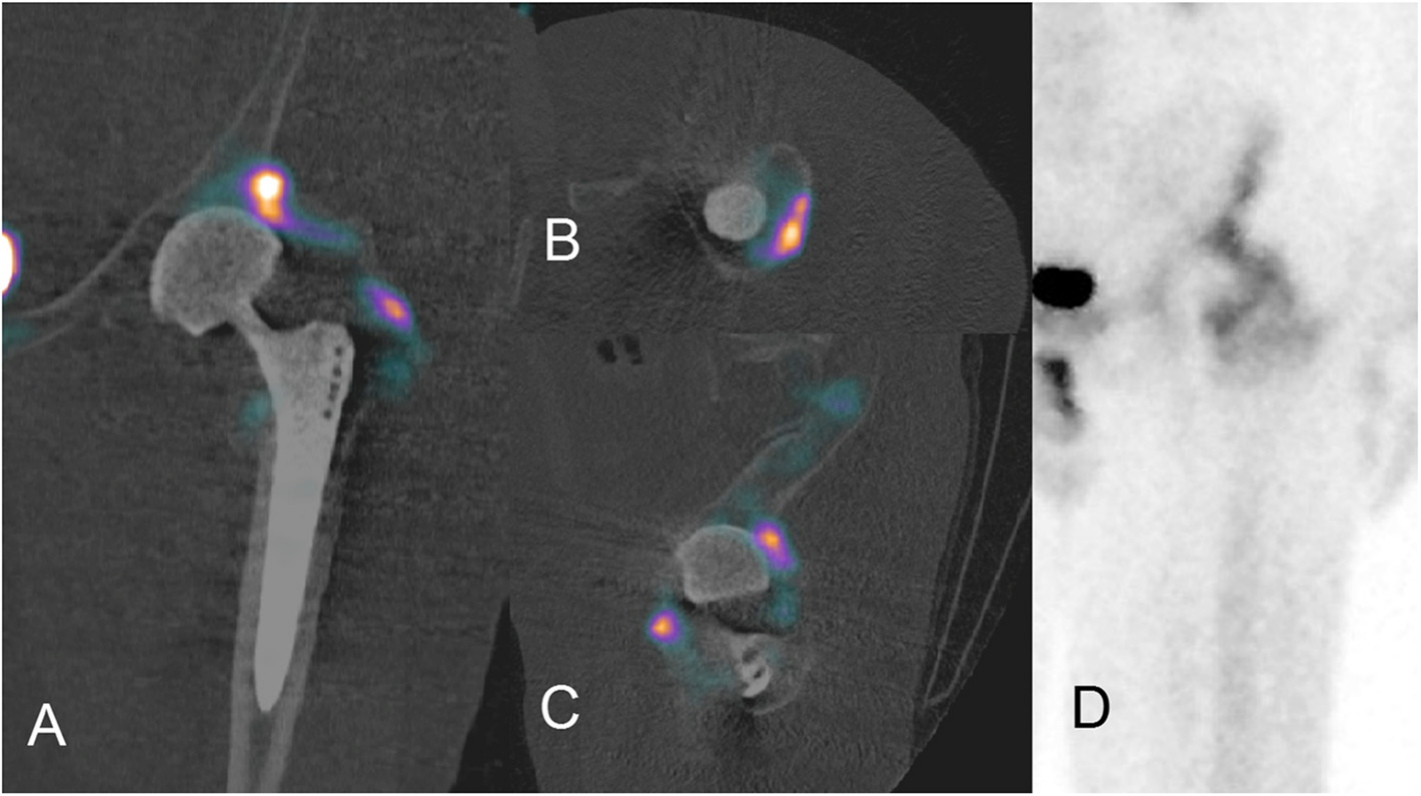
Differentiation between heterotopic ossifications and loosening of the acetabular component. 58-year old male with pain in the left hip that started several months after surgery. Using SPECT/CT, the increased uptake can be traced to heterotopic ossifications close to the acetabular component differentiating between loosening of the cup **A)**-**C)**. **D)** shows the planar SPECT image of the left hip with increased acitivity around the cup that cannot clearly be assigned to a specific structure. After a subsequent scintigraphic examination proving inactivity of the ossifications, these were removed surgically.

In 4 out of 18 patients the analysis of SPECT/CT data was superior to combined evaluation of planar scintigraphy, SPECT, and radiographs. In two patients SPECT/CT allowed the exclusion of prosthetic loosening, as the increased tracer uptake seen in planar scintigraphy and SPECT could be attributed to extra-articular heterotopic ossifications. (Figure 1.) In one patient the combination of a loosened cerclage placed for stabilization of a periprosthetic femoral shaft fracture and the consecutive loosening of the femoral prosthetic implant component was shown. Although the pathological tracer uptake would also have been discernible in conventional scintigraphy and SPECT, the causality and complexity of these interactive findings became only evident after SPECT/CT interpretation. In another patient SPECT/CT showed no pathology of the hip prosthesis, but revealed a symptomatic spinal stenosis.

In contrast to the planar setting with two false positive cases, the analysis with hybrid SPECT/CT correctly answered the question of prosthetic loosening in all cases (true positive, n = 6; true negative, n = 12).

## Discussion

This study evaluated BS with SPECT/CT for the diagnosis of unclear hip pain following uncemented total hip arthroplasty. Most causes of failure are detected through a the routine procedure including radiographs, clinical examination, patient history, CRP and ESR. [10] This study, on the other hand, assesses the potential of a relatively new technique in a highly selected group of patients that were not adequately diagnosed with the conventional methods. BS with SPECT is a very sensitive method for the detection of changes in bone-turnover and CT is the method of choice for structural bone imaging [11]. The combination of SPECT and CT was shown to provide an advantage over the interpretation of planar scintigraphic images and radiographs.

### Uptake pattern and CT interpretation

**Figure 2:**
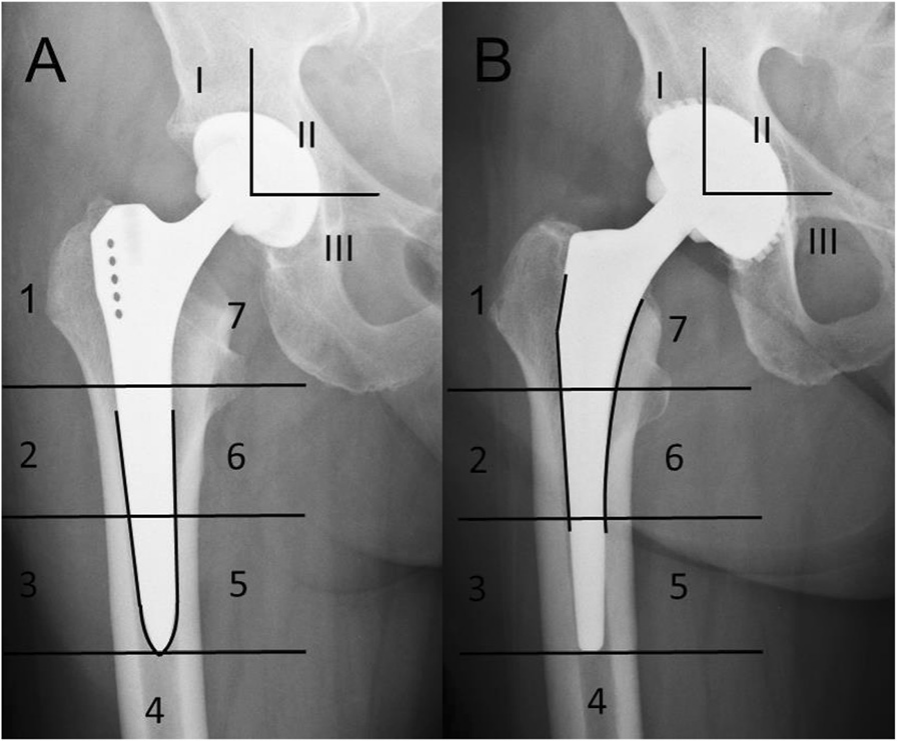
Gruen zones and crucial fixation zones of two standard stems in THA. **A)** Example of a diaphyseal or distal locking stem, crucial fixation zones marked with the black line (zones 2-6) **B)** Example of a stem with metaphyseal/diaphyseal fixation, crucial fixation zones marked with the black line (zones 1,2,6,7). Zones of the cup are numbered (I-III), I for the superior third, II for the intermediate third and III for the inferior third.

Interpretation of scintigraphic uptake is dependent on the biomechanical characteristics of each prosthesis. Uncemented implants rely on osteointegration, and increased tracer uptake around a hip prosthesis is observed postoperatively. Apart from the greater trochanter and the tip of the prosthesis (Gruen [12] zones 1 and 4, Figure 2), which may show increased tracer uptake for longer than 12 months, bone turnover assessed with BS is close to normal after one year [13,14]. For reliable interpretation, we still do not recommend scintigraphic assessment of an uncemented hip prosthesis earlier than 18 months after implantation.

Uncemented cups usually have their crucial fixation zones at the outer circumference e.g. the superior (I) and inferior third (III). (Figure 2) Loosening was diagnosed when increased tracer uptake was found at the prosthesis-bone interface in more than one compartment or if increased tracer uptake corresponded with a radiolucent line or osteolysis seen on CT-scan. Increased tracer uptake in the superior or inferior third not adjacent to the bone-prosthesis interface was a common finding and rated as physiological bone-remodelling [15]. The central third of the cup does not necessarily require contact to the bone and is not crucial for fixation, sometimes osteolysis is observed in this area but does not affect stability of the cup.

**Figure 3:**
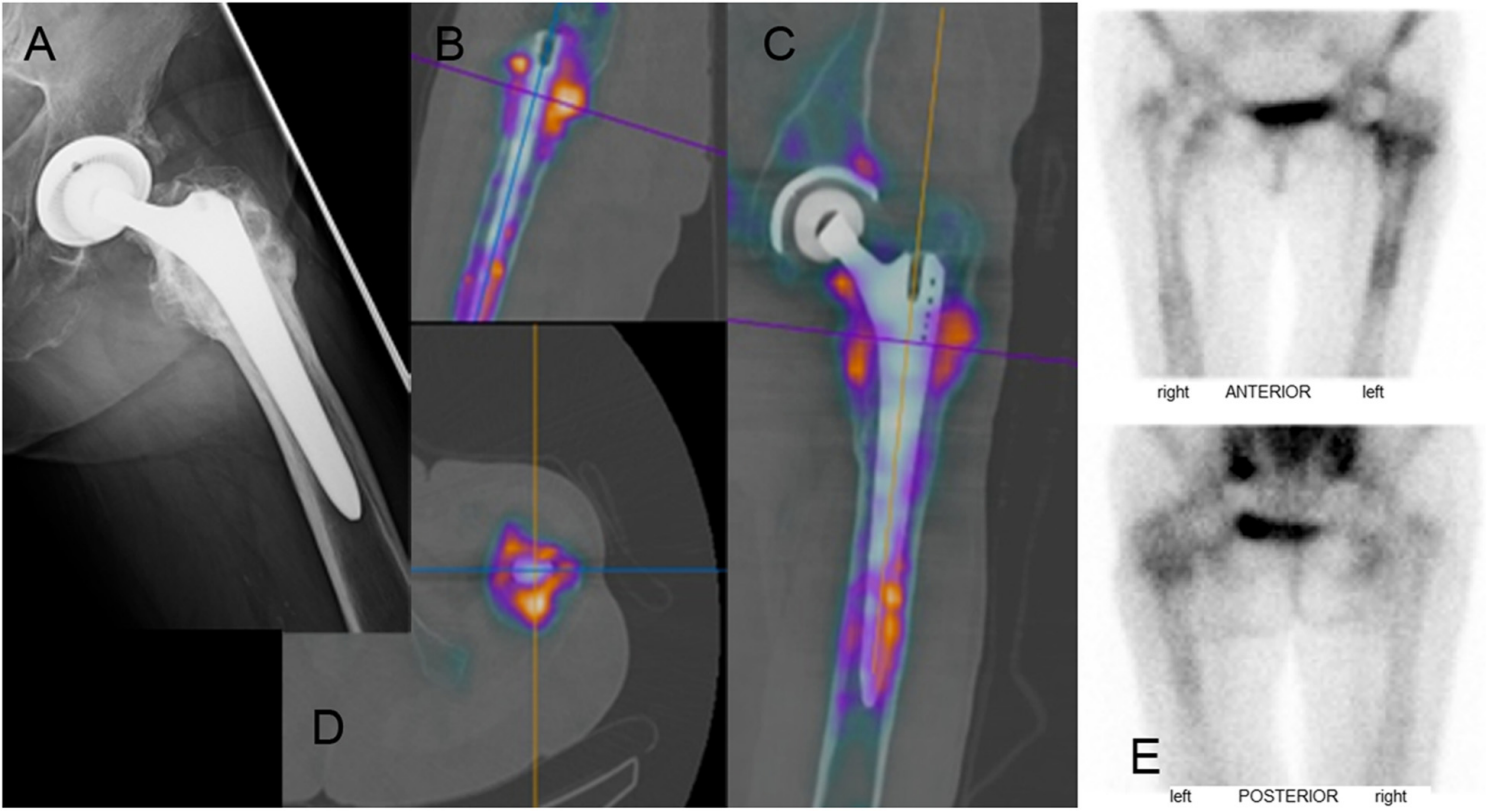
Example of pathological tracer uptake around a loosened stem. 80-year old female patient with THA of the left hip with a diaphyseal locking revision-stem. The patient had a history of several operations after a subtrochanteric femurfracture and complains now of therapy-resistant thigh-pain. **A)** shows the axial view of the left hip without definite signs of loosening. **B)**-**D)** shows pathological tracer uptake at the whole bone-prosthesis interface, consistent with the grading F3,1. The acetabular cup seen in **C)** shows isolated enhancement in the superior third, which was interpreted as physiological bone remodelling and graded A2 according to our classification. **E)** shows the anterior and posterior view of planar SPECT images of the pelvis. Intraoperative findings confirmed the diagnosis and the stem was exchanged.

**Figure 4:**
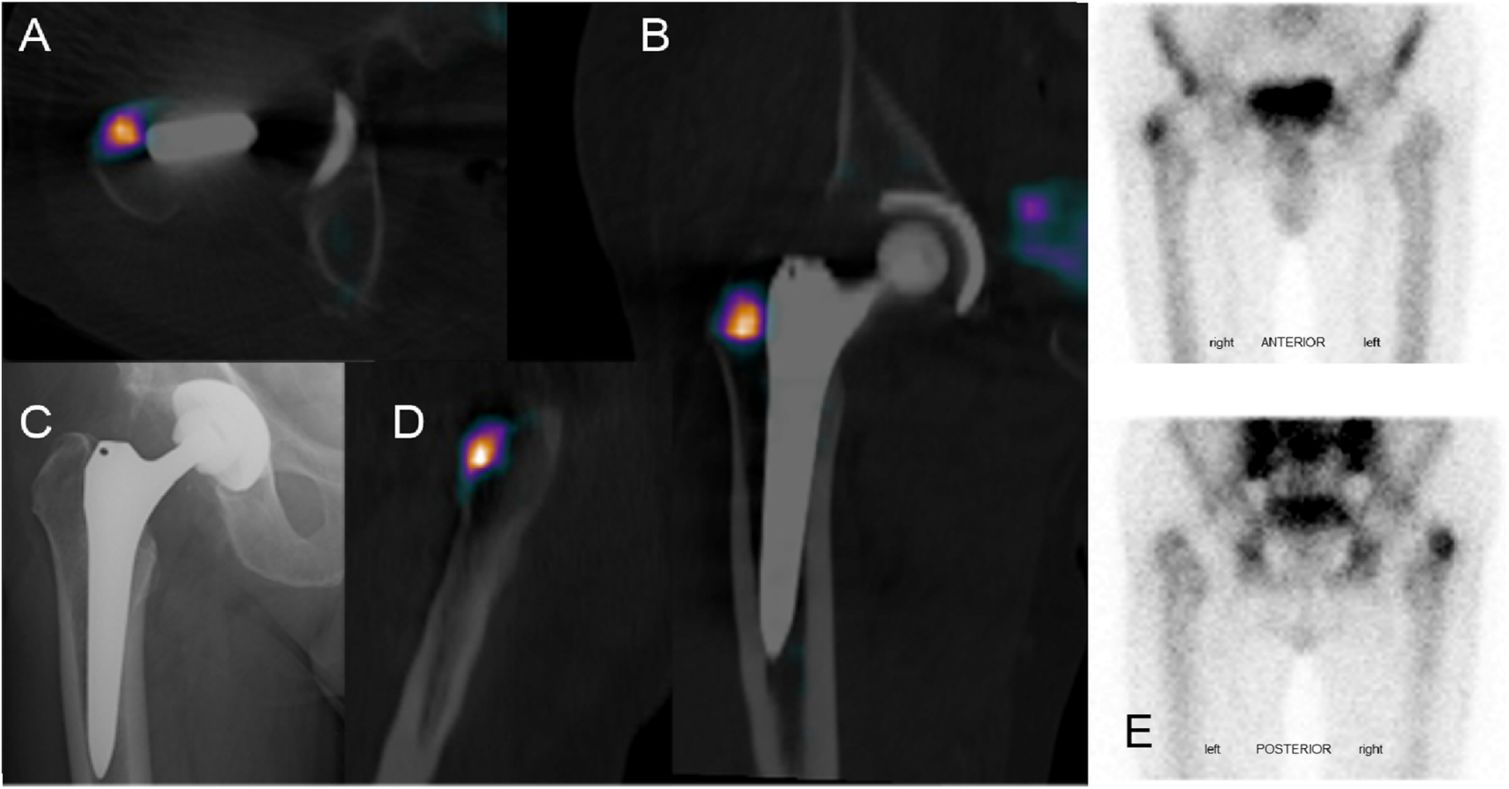
Example of physiological tracer uptake in THA. 77-year old male patient with a diaphyseal fixating stem of the right hip, suffering from pain in the right hip. **C)** shows the stem in varus position and a radiolucent line (stress shielding) in Gruen zone 1. **A**), **B**) and **D)** show increased bone metabolism in the greater trochanter. This represents physiological enhancement and is consistent with the grading F2,1. After further investigations a spine-related cause of the pain was diagnosed via MRI.

In this study-group three basic stem-types of total hip arthroplasty were examined: Stems with distal (diaphyseal) fixation (Figure 2A), stems with metaphyseal/diaphyseal fixation (Figure 2B) and short stems with proximal fixation. The crucial fixation zone of the distal locking stem is the cortical contact-zone in the diaphysis [16] (Gruen zones 2,3,5,6), radiolucency or increased tracer uptake at the bone-prosthesis interface in this area indicates loosening. (Figure 3) Increased uptake at the tip of the prosthesis only in combination with signs of loosening elsewhere was interpreted as axial movement of the stem and therefore also as loosening. Proximal radiolucency medial or lateral of the stem (Gruen zones 1,7), so-called stress shielding is observed frequently and does not indicate loosening. (Figure 4) Stems with diaphyseal fixation may show signs of loosening at the proximal end of the stem, due to slight movement or swinging of the prosthesis [17].

Stems with metaphyseal/diaphyseal fixation rely on osteointegration of the mid-section on the implant, which is often coated or of a porous surface to enhance osteointegration. Gruen zones 6 and 7, as well as the distal part of Gruen zones 1 and 2 represent the crucial fixation zone of these stems. Radiolucency along with increased tracer-uptake may be observed at the distal part and tip of the prosthesis despite a stable fixation of the prosthesis [18,19]. Increased tracer uptake and signs of loosening at the proximal prosthesis-bone interface may also be observed to a certain extent without loosening of the prosthesis.

Because of the various principles of fixation we propose a close cooperation with the orthopaedic surgeon responsible in any uncertain case, in order to provide an optimized report.

CT provides 3D-information of the pelvis, femur and acetabular and femoral component of THA. Thus exact positioning of the prosthesis with cup anteversion and stem antetorsion can be assessed. The published range of 10°-15° for femoral antetorsion according to Tönnis et al. and cup anteversion of 5°-25° according to Lewinnek et al. represent a so-called safe zone [20,21]. In one case stem antetorsion of 45° and cup anteversion of 35° were measured, but the case remains unclear as in this incidence of uncertain persisting pain the patient has not yet been willing to undergo re-operation. In addition CT also allows a planar and volumetric evaluation of polyethylene wear and osteolysis, thus providing significantly more information than planar radiographs [22].

CT also shows clear benefits in the detection of hardware failure over radiographs and the characteristics of computed tomography suggest a benefit in the diagnosis of loosening, though this remains to be proven in clinical studies [23,24].

### Causes of failure

Aseptic loosening is the most frequent cause for revision, followed by dislocation, infection and fracture [2]. Sensitivity and specificity of radiographs for the detection of loosening is 82% and 81% for the femoral component and 70% and 80% for the acetabular component [25,26]. In the clinical workflow periprosthetic infection is considered unlikely if serum CRP and ESR are not elevated [9]. Dislocation and periprosthetic fractures are usually diagnosed clinically and via radiograph. With the inclusion criteria of this study, the remaining study-group is highly selected and contains only complicated and challenging cases.

In this study 5 cases of hip pain were spine related and the respective region was examined via SPECT/CT after planar imaging had shown an increased tracer uptake. It should therefore be discussed whether a routinely performed SPECT-examination of the lumbar spine should be recommended and added to the imaging protocol.

Ossifications are a known cause for hip pain and limited range of motion after THA. Conservative treatment or radiation is usually ineffective and surgical resection is the only curative treatment [27,28]. To minimize the chance of reoccurrence, surgical excision should be performed after maturation of the lesion, which can be determined with BS [29]. Activity and exact location of ossifications can be assessed with SPECT/CT and can be differentiated from component loosening if increased tracer uptake is located around the acetabular cup or the greater trochanteric region. In this cohort 4/5 patients with heterotopic ossifications were successfully treated with surgical excision after component loosening had been ruled out with SPECT/CT.

Differentiating between septic and aseptic loosening is a challenge with no exact definition for a periprosthetic infection [30]. In accordance with current guidelines a periprosthetic infection in this study-group was considered to be unlikely after a blood test with negative CRP and ESR with a negative likelihood ratio of 0 to 0.06 [9]. However, periprosthetic joint infection can never be excluded with certainty. Especially low-grade infection is a major diagnostic challenge and is methodically not adequately assessed with bone SPECT/CT. Therefore infection should remain within the spectrum of differential diagnoses as long as sufficient invasive diagnostics have been performed. This is demonstrated by one case in this study group, where the final diagnosis was septic loosening. Initial blood tests showed no abnormalities and SPECT/CT indicated loosening of the stem (F3,2). The scintigraphic uptake-pattern in this case of septic loosening did not differ from aseptic loosening, including the blood flow and blood pool phases of triple phase BS. Other imaging modalities such as white blood cell imaging, anti-granulocyte scintigraphy or FDG-PET offer an alternative for the differentiation of septic from aseptic loosening of THA [31].

### Attenuation correction and radiation exposure

Attenuation artefacts due to metallic implants can be problematic for the assessment of bone turnover at the periphery of the implant using hybrid SPECT/CT images, as they may lead to an overestimation of pixel intensity after attenuation correction. Nevertheless, a recent study showed that a reliable and robust interpretation of bone SPECT/CT images of the femoral head-neck junction is feasible even in the presence of a metal hip resurfacing implant [32]. Besides the problem of artefacts introduced by CT-based attenuation correction, the work flow for the interpretation of integrated SPECT/CT examinations, especially in terms of CT-diagnostics, needs to be improved and implemented.

Radiation exposure in hybrid examinations is a limiting factor, but new acquisition protocols and recent methodological advances like iterative CT reconstruction present promising ways to reduce exposure to a minimum (apart from the reduction of attenuation correction artefacts).

The scan protocol resulted in a volume CT dose index (CTDIvol) of 2.2 mGy and a dose-length product (DLP) of 88 mGy*cm for a single bed position. This results in an effective dose E* of 1.1 mSv for a standard patient geometry [33]. However, exposure to ionizing radiation is nevertheless an important issue and an individual risk assessment must routinely be performed prior to every examination.

### Limitations

Limitations of this study are the retrospective character, the low number of patients enrolled and the lack of a uniform standard of reference.

Furthermore, it is costly and time consuming, and requires exposure to ionizing radiation.

## Conclusion

The study analysed the use of hybrid SPECT/CT for the evaluation of hip pain after THA. The results provide encouraging data of the benefits of SPECT/CT in a well-selected group of patients, especially where a mechanical or biomechanical problem is suspected. Hybrid SPECT/CT of THA reliably detects or rules out loosening and provides valuable information about the extent and maturity of heterotopic ossifications. Additionally, the CT component has the potential to assess mechanical failure, component alignment, osteolysis and fracture.

The whole-body scan provides information that may lead to the correct diagnosis apart from the suspected focus while a routinely executed SPECT of the lower spine may increase the sensitivity for pathologies in that area.

Future studies should prospectively evaluate the effectiveness of SPECT/CT and the developed grading system, specifically with regard to value in clinical work-flow, costs and technical optimization of the integrated imaging method. A CT-protocol that allows assessment of implant position, hardware failure, component fixation and abnormalities of bones or soft tissue needs to be meticulously integrated into the SPECT/CT-protocol in order to fully benefit from the advantages of hybrid imaging.

## Abbreviations

BS: Bone scintigraphy
CRP: C-reactive protein
CT: Computed tomography
ESR: Erythrocyte sedimentation rate
MRI: Magnetic resonance imaging
SPECT: Single photon emission computed tomography
SPECT/CT: Single photon emission computed tomography/computed tomography
Tc 99 m – DPD: Technetium-99 m dicarboxypropane diphosphonate
THR: Total hip replacement

## References

[1] Kurtz S, Mowat F, Ong K (2005). Prevalence of primary and revision total hip and knee arthroplasty in the United States from 1990 through 2002. J Bone Joint Surg Am.

[2] Annual Report of the Swedish Hip Arthroplasty Register 2011 (http://www.shpr.se/Libraries/Documents/%c3%85rsrapport_2011_eng_webb.sflb.ashx)

[3] Henderson RA, Lachiewicz PF (2012). Groin pain after replacement of the hip: aetiology, evaluation and treatment. J Bone Joint Surg Br.

[4] Borens O, Corvec S, Trampuz A (2012). Diagnosis of periprosthetic joint infections. Hip Int.

[5] Scharf S (2009). SPECT/CT imaging in general orthopedic practice. Semin Nucl Med.

[6] Huellner MW, Strobel K. Clinical applications of SPECT/CT in imaging the extremities. Eur J Nucl Med Mol Imaging. 2014 May;41 Suppl 1:S50-8. 10.1007/s00259-013-2533-5 23963296

[7] Tam HH, Bhaludin B, Rahman F, Weller A, Ejindu V, Parthipun A (2014). SPECT-CT in total hip arthroplasty. Clin Radiol.

[8] Graute V, Feist M, Lehner S, Haug A, Müller PE, Bartenstein P (2010). Detection of low-grade prosthetic joint infections using 99mTc-antigranulocyte SPECT/CT: initial clinical results. Eur J Nucl Med Mol Imaging.

[9] Filippi L, Schillaci O (2006). Usefulness of hybrid SPECT/CT in 99mTc-HMPAO-labeled leukocyte scintigraphy for bone and joint infections. J Nucl Med.

[10] Della Valle C, Parvizi J, Bauer TW, Dicesare PE, Evans RP, Segreti J (2010). American Academy of Orthopaedic Surgeons. Diagnosis of periprosthetic joint infections of the hip and knee. J Am Acad Orthop Surg.

[11] Linke R, Kuwert T, Uder M, Forst R, Wuest W (2010). Skeletal SPECT/CT of the peripheral extremities. AJR Am J Roentgenol.

[12] Gruen TA, McNeice GM, Amstutz HC (1979). “Modes of failure” of cemented stem-type femoral components: a radiographic analysis of loosening. Clin Orthop Relat Res.

[13] Kröger H, Vanninen E, Overmyer M, Miettinen H, Rushton N, Suomalainen O (1997). Periprosthetic bone loss and regional bone turnover in uncemented total hip arthroplasty: a prospective study using high resolution single photon emission tomography and dual-energy X-ray absorptiometry. J Bone Miner Res.

[14] Schmidt C, Born H (1990). The behavior of 3-phase scintigraphy of hip joint prostheses. Z Orthop Ihre Grenzgeb.

[15] Kwon YM, Higgs RJ, Bruce W (2001). Scintigraphic assessment of the acetabulum after arthroplasty with reference to cup geometry. Nucl Med Commun.

[16] Zweymüller K, Lintner F, Semlitsch M (1988). Biological fixation of a Titanium hip joint endoprosthesis. Clin Orthop.

[17] Harris WH (1992). Will stress shielding limit the longevity of cemented femoral components of total hip replacement?. Clin Orthop Relat Res.

[18] Engh CA, Massin P, Suthers KE (1990). Roentgenographic assessment of the biologic fixation of porous-surfaced femoral components. Clin Orthop Relat Res.

[19] Nakamura S, Arai N, Kobayashi T, Matsushita T (2012). Fixation of an anatomically designed cementless stem in total hip arthroplasty. Adv Orthop.

[20] Tönnis D, Heinecke A (1999). Acetabular and femoral anteversion: relationship with osteoarthritis of the hip. J Bone Joint Surg Am.

[21] Lewinnek GE, Lewis JL, Tarr R, Compere CL, Zimmerman JR (1978). Dislocations after total hip-replacement arthroplasties. J Bone Joint Surg Am.

[22] Chiang PP, Burke DW, Freiberg AA, Rubash HE (2003). Osteolysis of the pelvis: evaluation and treatment. Clin Orthop Relat Res.

[23] Ohashi K, El-Khoury GY, Bennett DL, Restrepo JM, Berbaum KS (2005). Orthopedic hardware complications diagnosed with multi-detector row CT. Radiology.

[24] Roth TD, Maertz NA, Parr JA, Buckwalter KA, Choplin RH (2012). CT of the hip prosthesis: appearance of components, fixation, and complications. Radiographics.

[25] Temmerman OP, Raijmakers PG, Berkhof J, Hoekstra OS, Teule GJ, Heyligers IC (2005). Accuracy of diagnostic imaging techniques in the diagnosis of aseptic loosening of the femoral component of a hip prosthesis: a meta-analysis. J Bone Joint Surg Br.

[26] Temmerman OP, Raijmakers PG, Deville WL, Berkhof J, Hooft L, Heyligers IC (2007). The use of plain radiography, subtraction arthrography, nuclear arthrography, and bone scintigraphy in the diagnosis of a loose acetabular component of a total hip prosthesis: a systematic review. J Arthroplasty.

[27] Neal B, Gray H, MacMahon S, Dunn L (2002). Incidence of heterotopic bone formation after major hip surgery. ANZ J Surg.

[28] Schauwecker J, Pohlig F, Toepfer A, Gollwitzer H, von Eisenhart-Rothe R (2011). Heterotopic ossifications in total hip arthroplasty: prophylaxis and therapy. Orthopade.

[29] Shehab D, Elgazzar AH, Collier BD (2002). Heterotopic ossification. J Nucl Med.

[30] Parvizi J (2011). New definition for periprosthetic joint infection. Am J Orthop (Belle Mead NJ).

[31] Ruf J, Oeser C, Amthauer H (2010). Clinical role of anti-granulocyte MoAb versus radiolabeled white blood cells. Q J Nucl Med Mol Imaging.

[32] Amarasekera HW, Roberts P, Costa ML, Parsons N, Achten J, Griffin DR (2012). Scintigraphic assessment of bone status at one year following hip resurfacing: Comparison of two surgical approaches using SPECT-CT scan. Bone Joint Res.

[33] Stamm G, Nagel HD (2002). [CT-expo--a novel program for dose evaluation in CT]. Rofo.

